# Optimization of SARS-CoV-2 M^pro^ Inhibitors by a Structure-Based Multilevel Virtual Screening Method

**DOI:** 10.3390/ijms26020670

**Published:** 2025-01-14

**Authors:** Lanlan Jing, Fabao Zhao, Lin Zheng, Bairu Meng, Shenghua Gao, Manon Laporte, Dirk Jochmans, Steven De Jonghe, Johan Neyts, Peng Zhan, Dongwei Kang, Xinyong Liu

**Affiliations:** 1 Key Laboratory of Chemical Biology (Ministry of Education), Department of Medicinal Chemistry, School of Pharmaceutical Sciences, Cheeloo College of Medicine, Shandong University, 44 West Culture Road, Jinan 250012, China; 2 Antiviral Drug & Vaccine Research Group, Department of Microbiology, Immunology and Transplantation, Rega Institute for Medical Research, KU Leuven, Herestraat 49, B-3000 Leuven, Belgium; 3 Molecular, Structural and Translational Virology Research Group, Department of Microbiology, Immunology and Transplantation, Rega Institute for Medical Research, KU Leuven, Herestraat 49, B-3000 Leuven, Belgium; 4 China-Belgium Collaborative Research Center for Innovative Antiviral Drugs of Shandong Province, Shandong University, 44 West Culture Road, Jinan 250012, China

**Keywords:** COVID-19, SARS-CoV-2, main protease, virtual screening

## Abstract

With the aim of developing novel anti-SARS-CoV-2 drugs to address the ongoing evolution and emergence of drug-resistant strains, the reported SARS-CoV-2 M^pro^ inhibitor **WU-04** was selected as a lead to find novel, highly potent, and broad-spectrum inhibitors. Using a fragment-based multilevel virtual screening strategy, 15 hit compounds were identified and subsequently synthesized. Among them, **A5** (IC_50_ = 1.05 μM), **A6** (IC_50_ = 1.08 μM), and **A9** (IC_50_ = 0.154 μM) demonstrated potent SARS-CoV-2 M^pro^ inhibition comparable to or slightly weaker than **WU-04**. Antiviral activity evaluations revealed that compound **A9** exhibited the strongest antiviral activity with an EC_50_ value of 0.18 μM, quite comparable to the marketed drug Nirmatrelvir (EC_50_ = 0.123 μM) and inferior to **WU-04** (EC_50_ = 0.042 μM). Molecular dynamics simulations elucidated the key interactions between compounds **A5**, **A6**, **A9**, and the binding pocket of SARS-CoV-2 M^pro^, providing valuable insights into their mechanisms of action. These findings identify compound **A9** as a promising lead for anti-SARS-CoV-2 drug development.

## 1. Introduction

Since 2019, the Severe Acute Respiratory Syndrome Coronavirus 2 (SARS-CoV-2), responsible for the Coronavirus Disease 2019 (COVID-19) pandemic, has presented an unparalleled global health emergency. As of 20 October 2024, the World Health Organization (WHO) reports 777 million confirmed cases and 7.1 million fatalities [1]. The incessant emergence of various SARS-CoV-2 variants such as Alpha, Beta, Gamma, Delta, and Omicron, characterized by heightened transmissibility and immune evasion, has significantly complicated pandemic control efforts [2]. While existing vaccines and antiviral therapies offer some protection and treatment against SARS-CoV-2, the continuous evolution of the virus and the emergence of resistance necessitate the development of novel antiviral strategies to combat current and future coronaviruses [3,4,5,6].

SARS-CoV-2 is a positive-sense single-stranded RNA virus which belongs to the β-coronavirus genus, along with SARS-CoV and MERS-CoV [7]. In the replication cycle of SARS-CoV-2, a key cysteine protease—namely the main protease (M^pro^, also known as Nsp5 and 3C-like protease)—is responsible for cleaving the pp1a and pp1ab polyproteins at 11 distinct sites, leading to the production of 12 non-structural proteins essential for viral replication [8,9,10]. The M^pro^ is highly conserved across various SARS-CoV-2 variants and shares a remarkable 96% sequence similarity with SARS-CoV M^pro^ [11]. Notably, M^pro^ cleaves polypeptide sequences exclusively after glutamine residues, a unique substrate specificity not shared by any human protease [12]. These distinctive features render M^pro^ a rational and safe target for the development of broad-spectrum anti-SARS-CoV-2 therapeutics.

**Figure 1:**
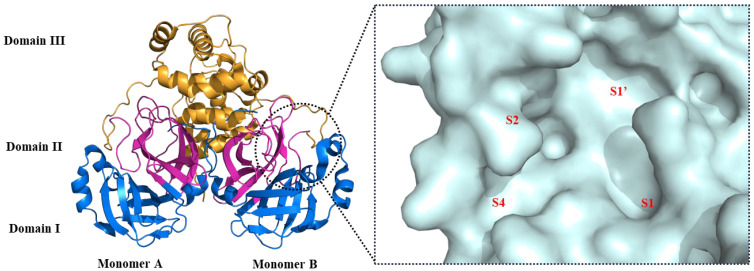
Crystal structure and catalytic pocket of SARS-CoV-2 M^pro^ (PDB code: 7EN8).

**Figure 2:**
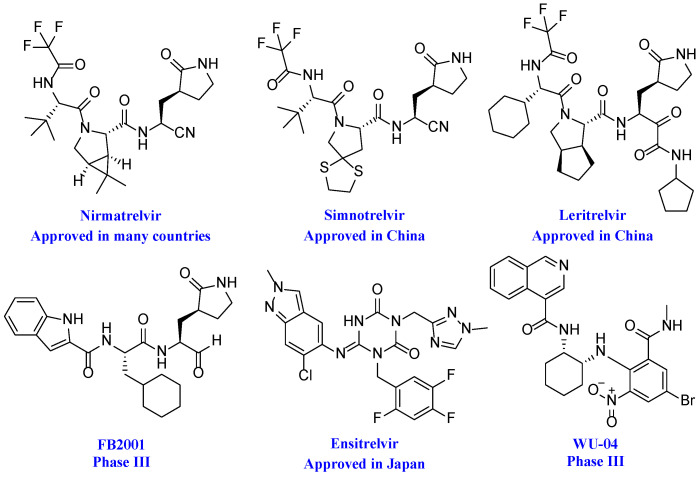
Representative SARS-CoV-2 M^pro^ inhibitors.

M^pro^ exists as a homodimer and each monomer consists of three domains (Figure 1). The protease catalytic cavity occupies the cleft between domains I and II. The active site of M^pro^ is formed by an active dyad Cys145-His41. The residue Cys145 that forms a C–S covalent bond with inhibitors is a key site in the substrate-binding pocket of M^pro^. Based on the interactions with its peptide substrates, the catalytic pocket of 3CLpro can be divided into four subsites: S1′, S1, S2, and S4 [13,14]. At present, many SARS-CoV-2 M^pro^ inhibitors have been approved or are undergoing clinical research (Figure 2), such as peptidomimetic covalent inhibitors Nirmatrelvir (**PF-07321332**) [15], Simnotrelvir (**SIM0417**) [16], Leritrelvir (**RAY1216**) [17,18], and **FB2001** [19] and non-covalent inhibitors Ensitrelvir (**S-217622**) [20] and **WU-04** [21]. However, peptidomimetic inhibitors of the SARS-CoV-2 M^pro^, such as Nirmatrelvir, have several drawbacks, including poor metabolic stability and the need for co-administration with cytochrome P450 inhibitors like ritonavir to prolong their exposure, potentially leading to drug interactions [22]. Additionally, their similar structures indicate a likelihood of inducing the same resistance mutations. Notably, multiple SARS-CoV-2 strains with M^pro^ mutations that confer resistance to nirmatrelvir have been discovered through in vitro studies, which may limit the effectiveness of these antiviral therapies over time [23,24]. **WU-04**, a novel non-covalent M^pro^ inhibitor identified by Hou et al., has demonstrated significant potency against the SARS-CoV-2 in the wild-type (EC_50_ = 10–25 nM), Delta (EC_50_ = 21 nM), and Omicron (EC_50_ = 24 nM) variants. It also effectively inhibits other coronaviruses, including SARS-CoV (EC_50_ = 19 nM) and MERS-CoV (EC_50_ = 53 nM) [21]. Currently, **WU-04** is in Phase III clinical trials (NCT06197217) [25].

**Figure 3:**
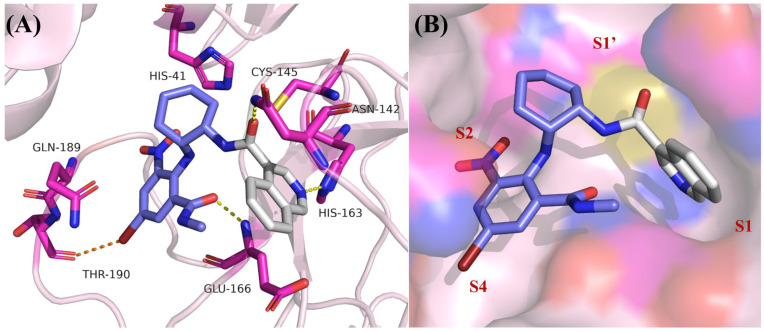
(**A**) The interactions of **WU-04** (light purple and gray) with the amino acid residues (magenta) surrounding the catalytic site of SARS-CoV-2 M^pro^, with hydrogen bond indicated by yellow dashed lines and halogen bond by orange dashed lines. (**B**) An overview of the catalytic pocket of the **WU-04**/M^pro^ complex.

The preliminary structure–activity relationship (SAR) analysis indicated that replacing the cyclohexane group at the core of **WU-04** with other nitrogen-containing heterocycles or bicyclic systems resulted in a significant loss or reduction in activity [21]. Similarly, the replacement of the 6-nitro and methylcarbamoyl groups on the bromophenyl ring also led to a significant decrease or even a complete loss of activity. The cocrystal structure of **WU-04** with M^pro^ provided further insights into its binding mode (Figure 3). Firstly, the isoquinoline ring, along with the 6-nitro and 4-bromo groups of the bromophenyl ring, occupied the S1, S2, and S4 pockets of the M^pro^ active site, respectively. Additionally, the cyclohexane-1,2-diamine group was critical for maintaining the conformation of **WU-04**. Secondly, the strong electron-withdrawing effect of the nitro group is essential for stabilizing the amino-π interaction between the phenyl ring and the side chain of Gln189, as well as for strengthening the halogen bond between the bromine atom and the carbonyl group of Thr190. Moreover, the carbonyl oxygen of the methylcarbamoyl group forms a hydrogen bond with the main chain amide of Glu166, further contributing to the compound’s binding affinity. In summary, the bromophenyl ring and the cyclohexane-1,2-diamine group of **WU-04** were critical for maintaining its activity. In addition, Jiang et al. also reported inhibitors with similar structures [26]. In contrast to **WU-04**, the isoquinoline ring was replaced by a quinolinone fragment, and modifications were made to the substituents at the 2- and 4-positions of the bromophenyl ring. The resulting compounds exhibited submicromolar antiviral activity.

Therefore, in this study, to further optimize the structure–activity relationship of the S1 pocket-targeting group in **WU-04** and enhance its antiviral activity, we retained the cyclohexane-1,2-diamine group and bromophenyl ring (depicted in light purple in Figure 3) as a core fragment. The isoquinoline group (depicted in gray in Figure 3) targeting the S1 pocket was substituted with various fragments, resulting in the construction of a novel and structurally diverse compound library. Through multilevel virtual screening and directed synthesis, 15 target compounds were obtained. These new compounds were subsequently characterized by enzymatic and antiviral activity evaluations, leading to the identification of three compounds displaying potent M^pro^ inhibition and antiviral efficacy.

## 2. Results

### 2.1. Fragment-Based Virtual Screening (FBVS)

**Figure 4:**
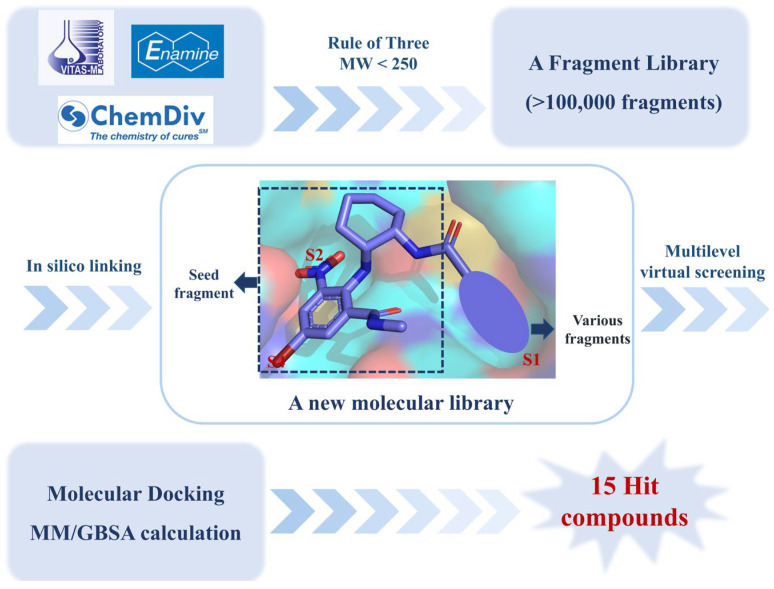
The discovery of hit compounds through fragment-based virtual screening.

Three databases (Vitas-M Laboratory, Enamine, and ChemDiv, over 500,000 fragments) were selected for the construction of the fragment library. Following previously reported methods [27], molecules that did not meet Rule of Three (RO3) [28] and those with a molecular weight exceeding 250 were excluded. This resulted in a fragment library comprising over 100,000 fragments. The selected fragments were then imported into the R-group database of Schrödinger suites. The initial structure of **WU-04** was prepared using the ligprep module. The isoquinoline ring was then removed and the left carbosyl motif was set as the connecting point of the diverse fragments. In silico linking was conducted using the R-group enumeration module in Schrödinger suites to systematically append the diverse fragments to the seed fragment, thereby constructing a novel molecule library (over 100,000 molecules). After that, the molecule library was prepared using the ligprep module. Subsequently, a multilevel virtual screening workflow was applied to the compound library focused on the M^pro^ active site (PDB code: 7EN8), which consists of a docking procedure at three accuracy levels, Glide HTVS (High-Throughput Virtual Screening), SP (Standard Precision), and XP (Extra Precision), as well as a binding free energy estimation based on MM-GB/SA free energy calculations. A total of 30 molecules were selected according to the multilevel virtual screening, and 15 of them were selected according to the synthetic feasibility analyses (Figure 4).

### 2.2. The Synthetic Route

**Scheme 1:**
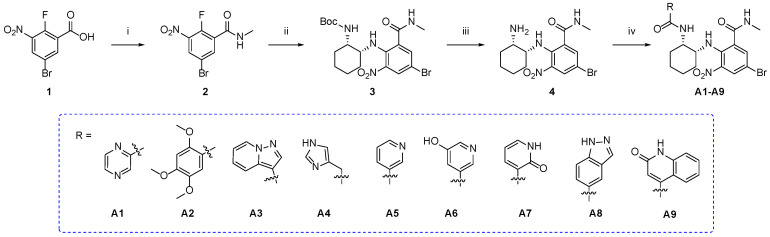
Synthesis of compounds **A1**–**A9**. Reagents and conditions: (i) CH_3_NH_2_·HCl, HATU, DIPEA, CH_2_Cl_2_, 0 °C; (ii) *tert*-butyl ((1*S*,2*R*)-2-aminocyclohexyl)carbamate, DIPEA, DMF, 80 °C; (iii) CF_3_COOH, CH_2_Cl_2_, r.t.; (iv) HATU, DIPEA, DMF, r.t.

**Scheme 2:**
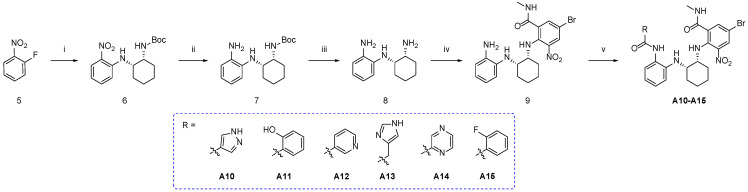
Synthesis of compounds **A10**–**A15**. Reagents and conditions: (i) *tert*-butyl ((1*R*,2*S*)-2-aminocyclohexyl)carbamate, DIPEA, DMF, 50 °C; (ii) Zn, NH_4_Cl, THF/H_2_O (3:2), r.t.; (iii) CF_3_COOH, CH_2_Cl_2_, r.t.; (iv) intermediate **2**, DIPEA, DMF, 50 °C; (v) HATU, DIPEA, CH_2_Cl_2_, r.t.

A total of 15 molecules (**A1**–**A15**) selected from the screening were synthesized. The synthetic route for compounds **A1**–**A9** is depicted in Scheme 1. Starting from 5-bromo-2-fluoro-3-nitrobenzoic acid (**1**), intermediate **2** was prepared via amide condensation, followed by nucleophilic substitution with *tert*-butyl ((1*S*,2*R*)-2-aminocyclohexyl)carbamate to yield intermediate **3**. The Boc protecting group was subsequently removed under trifluoroacetic acid (TFA) conditions to produce intermediate **4**, which was then condensed with various carboxylic acids affording target compounds **A1**–**A9**. The synthesis of compounds **A10**–**A15** is illustrated in Scheme 2. The starting material, 1-fluoro-2-nitrobenzene (**5**), was reacted with *tert*-butyl ((1*R*,2*S*)-2-aminocyclohexyl)carbamate via nucleophilic substitution to obtain intermediate **6**. Reduction of intermediate **6** yielded intermediate **7**, which was then deprotected by removing the Boc group to afford intermediate **8**. Finally, target compounds **A10**–**A15** were synthesized through nucleophilic substitution and amide condensation reactions.

### 2.3. Enzyme Inhibitory Activities

**Figure 5:**
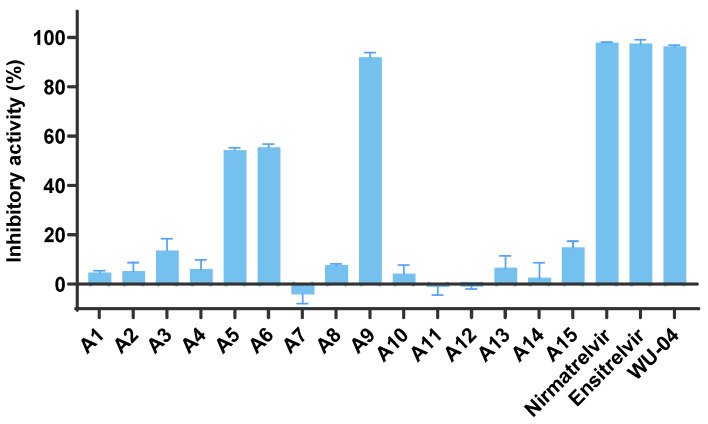
SARS-CoV-2 M^pro^ inhibitory activity in the presence of **A1**–**A15** (at 1 μM).

All newly synthesized compounds (**A1**–**A15**) were initially tested for their inhibitory activity against SARS-CoV-2 M^pro^ using a fluorescence resonance energy transfer (FRET)-based enzymatic assay at a single concentration of 1 μM. Nirmatrelvir, Ensitrelvir, and **WU-04** were used as reference compounds. Compounds **A5**, **A6**, and **A9** showed potent inhibitory activity, with inhibition rates of 53.7%, 55.5%, and 92.0%, respectively, while the inhibitory activity of other compounds was significantly less (Figure 5). The generation of dose–response curves allowed us to determine IC_50_ values for compounds **A5**, **A6**, and **A9**. Compound **A9** showed the strongest inhibitory activity with an IC_50_ value of 0.154 μM, which is comparable to that of lead compound **WU-04** (IC_50_ = 0.125 μM), as well as marketed drugs Nirmatrelvir (IC_50_ = 0.103 μM) and Ensitrelvir (IC_50_ = 0.113 μM). Compounds **A5** (IC_50_ = 1.05 μM) and **A6** (IC_50_ = 1.08 μM) also showed strong inhibitory activity against SARS-CoV-2 M^pro^, although it was slightly inferior to that of **WU-04**, Nirmatrelvir, and Ensitrelvir (Table 1).

### 2.4. Anti-SARS-CoV-2 Activity

The antiviral activity of compounds with potent M^pro^ inhibition was tested in VeroE6-eGFP cells infected with SARS-CoV-2 (BetaCov/Belgium/GHB-03021/2020 B.1 lineage A), and cytotoxicity was also assessed using established methods [29,30]. **WU-04** and Nirmatrelvir were used as positive controls. The results are depicted in Table 2. All compounds demonstrated antiviral activity in the micromolar or submicromolar range. Notably, compound **A9** exhibited the most potent antiviral activity from this series, with an EC_50_ value of 0.18 μM, comparable to that of Nirmatrelvir (EC_50_ = 0.123 μM) but slightly less potent than **WU-04** (EC_50_ = 0.042 μM). Compounds **A5** and **A6** were clearly less potent, with EC_50_ values ranging from 2.01 to 6.05 μM. The results obtained from both enzymatic activity assays and cell-based antiviral assays exhibit a high degree of concordance. For instance, compound **A9**, which showed the highest enzymatic inhibitory activity, also displayed the strongest antiviral activity. In addition, none of the compounds exhibited significant cytotoxicity at a concentration of 100 μM. These findings suggest that the fragment-based virtual screening strategy is a rational and feasible approach for discovering highly potent antiviral agents against SARS-CoV-2.

### 2.5. Molecular Modeling Analysis

**Figure 6:**
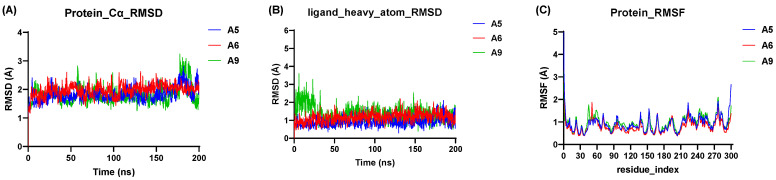
(**A**) The Cα RMSD plot of the SARS-CoV-2 M^pro^ in complex with ligands **A5**, **A6**, or **A9** during a 200 ns MD simulation. (**B**) The heavy atom RMSD plot of ligands **A5**, **A6**, or **A9** when bound to the SARS-CoV-2 M^pro^ over a 200 ns MD simulation. (**C**) The RMSF plot of the protein chain in the bound states of **A5**, **A6**, or **A9**.

Molecular docking studies were performed using the induced-fit docking module of Schrödinger Suite software (Version 2023-1, Schrödinger, New York, NY, USA) to shed a light on the binding modes of representative compounds **A5**, **A6**, and **A9** with SARS-CoV-2 M^pro^. The cocrystal structures of SARS-CoV-2 M^pro^ (PDB code: 7EN8) were used as input structures for docking calculations. The molecular dynamics simulations (MD) were performed for 200 ns using Desmond to further investigate the stability of representative compounds with SARS-CoV-2 M^pro^. The root mean square deviation (RMSD) and root mean square fluctuation (RMSF) of simulation trajectories were plotted (Figure 6). The RMSD values of the protein Cα atoms and ligand heavy atoms remained relatively constant throughout the simulation, indicating stable binding of compounds **A5**, **A6**, and **A9** to the SARS-CoV-2 M^pro^ (Figure 6A,B). The analysis of root mean square fluctuation (RMSF) revealed that all ligand–protein complexes exhibited similar patterns of atomic fluctuations, suggesting a consistent interaction profile across the tested compounds (Figure 6C).

**Figure 7:**
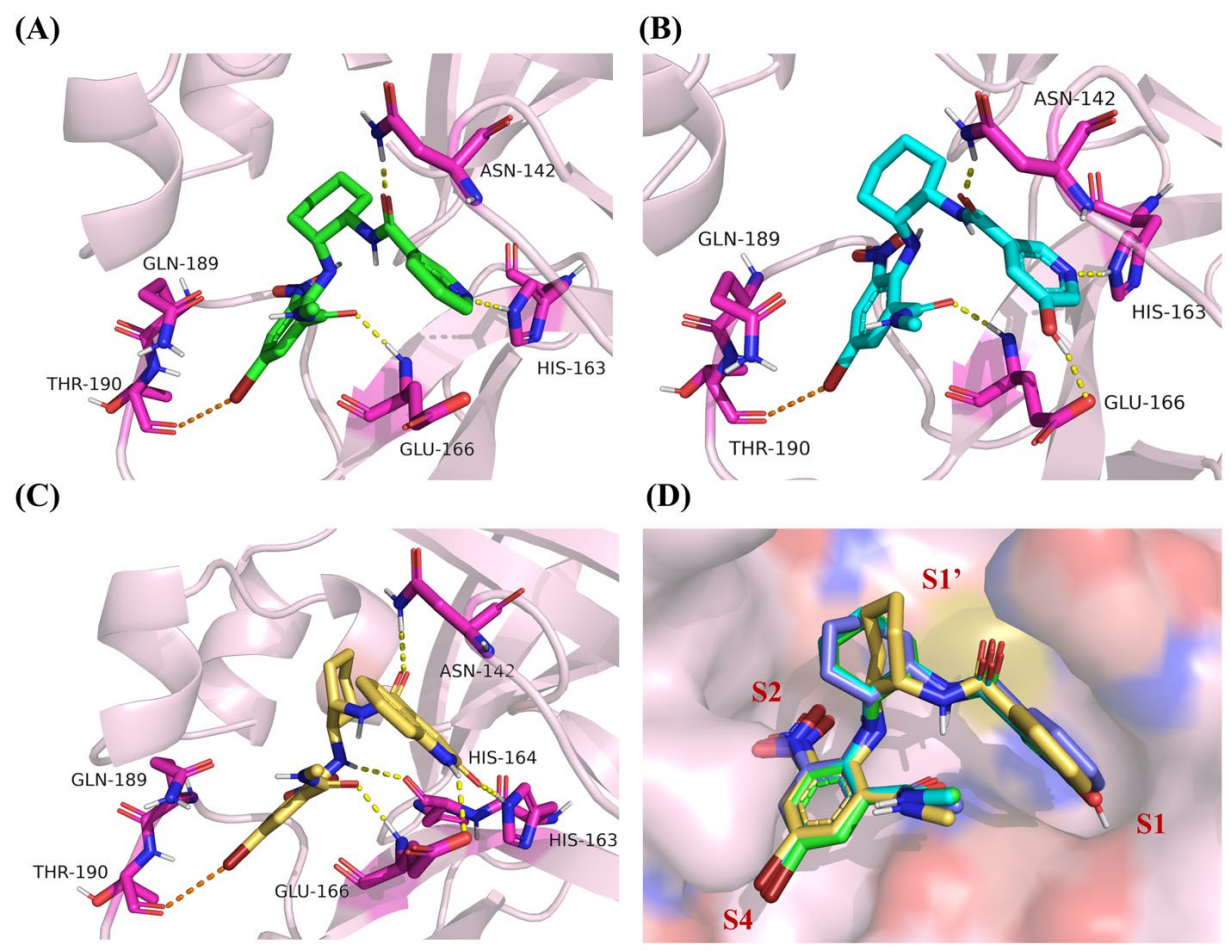
Predicted binding modes of representative compounds with SARS-CoV-2 M^pro^ (PDB code: 7EN8). (**A**) Detailed interactions between **A5** and residues surrounding the catalytic pocket of SARS-CoV-2 M^pro^. (**B**) Detailed interactions between **A6** and residues surrounding the catalytic pocket of SARS-CoV-2 M^pro^. (**C**) Detailed interactions between **A9** and residues surrounding the catalytic pocket of SARS-CoV-2 M^pro^. (**D**) Superimposition of the crystal structure of the SARS-CoV-2 M^pro^/WU-04 complex with predicted binding modes of representative compounds **A5**, **A6**, and **A9**. Color code: light purple, **WU**-**04**; green stick, **A5**; cyan stick, **A6**; yellow stick, **A9**; magenta, residues; yellow dash line: hydrogen bonds; orange dash line, halogen bond.

The most abundant binding conformation was clustered from the molecular dynamics simulations trajectory, and the results were visualized using PyMOL software (Version 2.6.0a0, Schrödinger, New York, NY, USA) and illustrated in Figure 7. The conformations of compounds **A5**, **A6**, and **A9** in the binding pocket are highly consistent with **WU-04**, as demonstrated by Figure 7D. These compounds occupied the S1, S2, and S4 pockets and maintained key hydrogen bond interactions. Specifically, the carbonyl oxygen atom connected to the phenyl ring formed a hydrogen bond with the NH group of Glu166, and the carbonyl oxygen atom of the acyl group connected to the cyclohexane ring maintained a hydrogen bond with Asn142. The pyrimidine nitrogen atoms of **A5** (Figure 7A) and **A6** (Figure 7B), as well as the quinolone amino group of **A9** (Figure 7C), formed hydrogen bonds with His163. Additionally, the hydroxyl group connected to the pyrimidine ring of **A6** and the carbonyl oxygen of the quinolone of **A9** formed additional hydrogen bonds with Glu166. Notably, the 1-NH group of the bromophenyl ring of **A6** formed an additional hydrogen bond with His164. This was not observed in other molecules and might be responsible for its more potent enzyme inhibitory activity.

Employing the MM-GBSA methodology, the absolute binding free energy of SARS-CoV-2 M^pro^ in complex with **A5**, **A6**, and **A9** was determined. In addition to the overall binding free energy, the representative contributions from different energetic components were delineated, including Coulomb energy (Coulomb), hydrogen-bonding correction (Hbond), lipophilic energy (Lipo), and Van der Waals energy (vdW). The data presented in Table 3 reveal that **A9** possessed the most negative total energy value, implying a more robust binding affinity to SARS-CoV-2 M^pro^ compared to **A5** and **A6**. Specifically, in contrast to the monocyclic structure of **A5** and **A6**, **A9** incorporated a larger quinolone scaffold in the region targeting the S1 pocket. This structural modification allowed **A9** to more effectively occupy the S1 pocket, thereby facilitating extensive hydrophobic interactions and Van der Waals forces with the surrounding amino acids. As a result, **A9** exhibited the most negative Coulomb energy (−26.04 kcal/mol), lower lipophilic energy (−13.98 kcal/mol) and Van der Waals energy (−61.38 kcal/mol). Notably, **A9** exhibited a significantly lower Hbond value, suggesting an enhanced hydrogen bonding interaction with the SARS-CoV-2 M^pro^, aligning with the docking results previously discussed. These results provide further insight into the rationale behind the superior inhibitory activity of **A9** against the SARS-CoV-2 M^pro^.

## 3. Discussion

To discover novel and potent small-molecule inhibitors of SARS-CoV-2 M^pro^, we selected **WU-04** as the lead compound, utilizing its cyclohexane-1,2-diamine group and bromophenyl ring as a seed fragment and replacing its isoquinoline moiety with structurally diverse fragments obtained through the screening of commercial fragment libraries, thus generating a novel small-molecule library. This library was subjected to multilevel virtual screening. The top 30 compounds were evaluated for synthetic feasibility, from which 15 compounds were selected for directed synthesis. Enzymatic inhibition assays of these 15 analogues revealed that compounds **A5** (IC_50_ = 1.05 μM), **A6** (IC_50_ = 1.08 μM), and **A9** (IC_50_ = 0.154 μM) exhibited significant inhibitory activities, comparable to or slightly weaker than the lead compound **WU-04** (IC_50_ = 0.125 μM) and marketed drugs Nirmatrelvir (IC_50_ = 0.103 μM) and Ensitrelvir (IC_50_ = 0.113 μM). The antiviral assays demonstrated that compounds **A5**, **A6**, and **A9** exhibited antiviral activities in the micromolar or submicromolar range, with compound **A9** (EC_50_ = 0.18 μM) showing potent activity comparable to Nirmatrelvir (EC_50_ = 0.123 μM) but slightly inferior to **WU-04** (EC_50_ = 0.042 μM). Molecular modeling provided insights into the interaction mechanisms between the representative compounds and the binding pocket of SARS-CoV-2 M^pro^.

Interestingly, we found that compound **A9** was recently also identified by Jiang et al. [26]. However, the design and methodology employed in their study are significantly different from those utilized in ours. First, unlike the traditional target-based structural modification strategy employed by Jiang et al. with **JZD-07** as the lead, our study utilized **WU-04** as the lead and applied a fragment library construction approach integrated with multilevel virtual screening. This innovative methodology facilitated the identification of structurally diverse and entirely novel small molecules. Second, they assessed the inhibitory activity of **A9** against SARS-CoV-2 M^pro^ and demonstrated that **A9** exhibited potent enzymatic inhibition with an IC_50_ value of 40.8 nM. In contrast to our study, they utilized MCA-AVLQSGFR-Lys(Dnp)-Lys-NH2 as the fluorescently labeled substrate. Furthermore, they evaluated the antiviral activity of **A9** against SARS-CoV-2 delta strains in Vero E6 cells by quantifying viral copy numbers in the cell supernatant via quantitative real-time PCR (qRT-PCR). Their results reveal that **A9** exhibited potent activity against the delta strain, with an EC_50_ value of 0.849 μM. However, they did not simultaneously test the antiviral activity of approved drugs under the same conditions, making it challenging to compare **A9**’s activity directly with other compounds. In contrast, our study employed a different testing method and evaluated both **A9** and the approved drug Nirmatrelvir under identical conditions. This provides a robust complement to the existing activity data for **A9**. Nevertheless, these findings support the feasibility and rationale of our fragment-based, multilevel virtual screening strategy for discovering novel and potent SARS-CoV-2 inhibitors.

Furthermore, this study offers a valuable framework for discovering new small-molecule antiviral inhibitors. However, several aspects of our approach warrant improvement to broaden its applicability. First, the diversity of the fragment library was limited, as we only included three major commercial databases: Vitas-M Laboratory, Enamine, and ChemDiv. Expanding the selection of fragment libraries in future studies could significantly enhance the molecular diversity of the target compounds. Second, while we employed molecular docking and MM-GB/SA calculations to identify compounds with high binding affinities, additional prioritization based on drug-likeness properties—such as toxicity, solubility, and metabolic stability—would likely improve the likelihood of identifying viable drug candidates. Addressing these limitations will be a key focus of our future work.

## 4. Materials and Methods

### 4.1. Construction of Compound Library and Virtual Screening

The crystal structure (PDB ID: 7en8) was prepared using the Protein Preparation Wizard in the Schrödinger suites (Release 2023-1, Schrödinger, New York, NY, USA). The protein bond order was reassigned with the CCD database. Protein bond orders were reassigned based on the CCD database, hydrogen atoms were added, and water molecules were removed. The protonation states of ligands and receptors were adjusted to pH 7.0, and missing loops and side chains were reconstructed using Prime. Restrained minimization was performed with the OPLS4 force field, and the resulting protein structure was used for subsequent steps.

The focused compound library was constructed using the R-group enumeration module. The binding pocket position was determined using the primary ligands from the crystal structure and defined through the grid generation module. Screening was conducted with the newly constructed small-molecule compound library, which had been prepared using LigPrep in Schrödinger Suite. Virtual screening was performed using the default parameters of the virtual screening workflow. The top 1000 ligands, based on docking scores, were selected, and their binding affinities were evaluated through post-MM-GBSA analysis. The ligands were selected manually, considering the docking scores, MM-GBSA binding affinity estimations, and the feasibility of their synthesis.

### 4.2. Chemistry

The solvents and reagents used in this study were commercially sourced and did not require any preprocessing before use. ^1^H and ^13^C NMR spectra were acquired in DMSO-*d*_6_ on a Bruker AV-400 spectrometer (Bruker Corporation, Billerica, MA, USA) with tetramethylsilane as the internal reference. Chemical shifts were denoted in ppm. The ^1^H NMR spectra data were reported as follows: chemical shift (δ) in ppm, multiplicity (s = singlet, d = doublet, t = triplet, q = quartet, m = multiplet), coupling constants (J) in hertz (Hz), and integration values (Figures S1–S6). Mass spectrometry of the compounds was conducted on a Thermo Fisher^TM^ LCQ Fleet ion trap mass spectrometer (Thermo Fisher Scientific, Waltham, MA, USA). Melting points were assessed with a micro melting point apparatus (RY-1G, TianGuang Optical Instruments, Tianjin, China). Reaction progress was tracked via thin-layer chromatography (TLC), with spots detected using iodine vapor or UV illumination. Rapid purification was achieved through flash column chromatography on silica gel (200–300 mesh, Haiyang Chemical Company, Qingdao, China). Purity of the compounds was ascertained by high-performance liquid chromatography (HPLC, Agilent Technologies, Santa Clara, CA, USA) equipped with a 5 μm C18 column (150 mm × 4.6 mm). The purity of all target compounds exceeded 95%.

5-Bromo-2-fluoro-N-methyl-3-nitrobenzamide (**2**)

5-Bromo-2-fluoro-3-nitrobenzoic acid (**1**) (2.0 g, 7.56 mmol) was dissolved in 20 mL of CH_2_Cl_2_ at 0 °C. Sequentially, CH_3_NH_2_·HCl (0.62 g, 9.07 mmol), 2-(7-azabenzotriazol-1-yl)-*N*,*N*,*N*′,*N*′-tetramethyluronium hexafluorophosphate (HATU) (4.31 g, 4.31 mmol), and *N*,*N*-diisopropylethylamine (DIPEA) (2.93 g, 22.68 mmol) were added. The reaction mixture was stirred at 0 °C for 3 h (monitored by TLC). Afterward, 30 mL of water was added to the reaction mixture, which was then extracted with CH_2_Cl_2_ (3 × 20 mL). The organic phase was washed with saturated sodium chloride (3 × 30 mL) and dried over anhydrous sodium sulfate to yield the crude product, which was purified by flash column chromatography on silica gel to obtain the intermediate **2**. White powder, 85% yield, mp: 140–142 °C. ^1^H NMR (400 MHz, DMSO-*d*_6_) δ 8.66 (d, *J* = 5.2 Hz, 1H), 8.45 (dd, *J* = 6.2, 2.6 Hz, 1H), 8.13 (dd, *J* = 5.2, 2.6 Hz, 1H), 2.79 (d, *J* = 4.6 Hz, 3H, CH_3_).

2-(((1*R*,2*S*)-2-Aminocyclohexyl)amino)-5-bromo-*N*-methyl-3-nitrobenzamide (**4**)

Intermediate **2** (1.0 g, 3.61 mmol), *tert*-butyl ((1*S*,2*R*)-2-aminocyclohexyl)carbamate (0.93 g, 4.33 mmol), and DIPEA (1.4 g, 10.83 mmol) were added to DMF and stirred at 80 °C for 12 h. The reaction was quenched by the addition of 30 mL of water, followed by extraction with CH_2_Cl_2_ (3 × 20 mL). The organic phases were combined, washed with saturated sodium chloride (3 × 30 mL), and dried over anhydrous sodium sulfate to yield the crude product **3**. The crude product 3 was then dissolved in 10 mL of CH_2_Cl_2_, to which 3 mL of trifluoroacetic acid was added. After stirring at room temperature for 2 h, the pH of the reaction mixture was adjusted to 8–9 using saturated sodium bicarbonate solution. The mixture was extracted with CH_2_Cl_2_ (3 × 20 mL), and the organic phase was washed with saturated sodium chloride (3 × 30 mL) and dried over anhydrous sodium sulfate to obtain the crude product, which was then purified by flash column chromatography on silica gel to yield intermediate **4**. Yellow powder, 66% yield, mp: 70–72 °C. ESI-MS: *m*/*z* 371.88 (M + H)^+^, 373.83 (M + 3H)^+^, C_14_H_19_BrN_4_O_3_ (370.06). ^1^H NMR (400 MHz, DMSO-*d*_6_) δ 8.69 (s, 1H), 8.56 (s, 1H), 8.18 (d, *J* = 2.5 Hz, 1H), 7.58 (d, *J* = 2.6 Hz, 1H), 2.93–2.83 (m, 1H), 2.76 (s, 3H, CONHCH_3_), 2.58–2.52 (m, 1H), 1.96–1.65 (m, 2H), 1.59–1.20 (m, 8H), 1.18–1.02 (m, 1H).

General synthesis procedure for compounds **A1**–**A9**.

A solution of various carboxylic acids (1.2 eq), HATU (1.5 eq), and DIPEA (2.0 eq) was prepared in DMF. The mixture was stirred at 0 °C for 30 min, after which intermediate 4 (1.0 eq) was added. The reaction mixture was then stirred at room temperature for 3 to 8 h. The reaction was terminated by the addition of 30 mL of water, followed by extraction with CH_2_Cl_2_ (3 × 20 mL). The organic phases were combined, washed with saturated sodium chloride (3 × 30 mL), and dried over anhydrous sodium sulfate to yield the crude product. Subsequent purification by flash column chromatography on silica gel afforded the target compounds **A1**–**A9**.

*N*-((1*S*,2*R*)-2-((4-Bromo-2-(methylcarbamoyl)-6-nitrophenyl)amino)cyclohexyl)pyrazine-2-carboxamide (**A1**)

Yellow powder, 82% yield, mp: 98–100 °C. ESI-MS: *m*/*z* 477.70 (M + H)^+^, 479.21 (M + 3H)^+^, C_19_H_21_BrN_6_O_4_ (476.08). ^1^H NMR (400 MHz, DMSO-*d*_6_) δ 9.06 (d, *J* = 1.5 Hz, 1H), 8.88 (d, *J* = 2.5 Hz, 1H), 8.69 (dd, *J* = 2.5, 1.5 Hz, 1H), 8.67 (d, *J* = 4.7 Hz, 1H), 8.30 (d, *J* = 9.0 Hz, 1H), 8.07 (d, *J* = 2.4 Hz, 1H), 7.98 (d, *J* = 10.5 Hz, 1H), 7.67 (d, *J* = 2.5 Hz, 1H), 4.24–4.11 (m, 1H), 3.71–3.58 (m, 1H), 2.72 (d, *J* = 4.6 Hz, 3H), 1.76–1.58 (m, 5H), 1.56–1.33 (m, 3H). ^13^C NMR (100 MHz, DMSO-*d*_6_) δ 167.32, 162.93, 148.05, 144.78, 143.88, 143.67, 142.52, 138.10, 137.26, 130.00, 128.35, 106.88, 55.07, 49.35, 28.41, 28.08, 26.75, 22.10.

5-Bromo-*N*-methyl-3-nitro-2-(((1*R*,2*S*)-2-(2,4,5-trimethoxybenzamido)cyclohexyl)amino)benzamide (**A2**)

Yellow powder, 81% yield, mp: 94–96 °C. ESI-MS: *m*/*z* 565.73 (M + H)^+^, 567.14 (M + 3H)^+^, C_24_H_29_BrN_4_O_7_ (564.12). ^1^H NMR (400 MHz, DMSO-*d*_6_) δ 8.67 (q, *J* = 4.6 Hz, 1H), 8.17–7.99 (m, 3H), 7.67 (d, *J* = 2.5 Hz, 1H), 7.34 (s, 1H), 6.75 (s, 1H), 4.18 (s, 1H), 3.90 (s, 3H), 3.86 (s, 3H), 3.73 (s, 3H), 3.60 (d, *J* = 9.5 Hz, 1H), 2.74 (d, *J* = 4.5 Hz, 3H), 1.77–1.31 (m, 8H). ^13^C NMR (100 MHz, DMSO-*d*_6_) δ 167.47, 164.30, 152.88, 152.69, 143.03, 142.52, 137.70, 137.32, 130.00, 114.07, 112.84, 106.57, 98.23, 57.40, 56.37, 56.31, 55.20, 40.57, 40.36, 40.16, 39.95, 39.74, 39.53, 39.32, 29.08, 28.38, 26.76.

*N*-((1*S*,2*R*)-2-((4-bromo-2-(methylcarbamoyl)-6-nitrophenyl)amino)cyclohexyl)pyrazolo [1,5-a]pyridine-3-carboxamide (**A3**)

Yellow powder, 79% yield, mp: 146–116 °C. ESI-MS: *m*/*z* 515.48 (M + H)^+^, 517.22 (M + 3H)^+^, C_22_H_23_BrN_6_O_4_ (514.10).^1^H NMR (400 MHz, DMSO-*d*_6_) δ 8.78–8.71 (m, 1H), 8.62–8.56 (m, 1H), 8.54 (s, 1H), 8.11 (d, *J* = 10.5 Hz, 1H), 8.09–8.03 (m, 2H), 7.68 (d, *J* = 8.3 Hz, 1H), 7.61 (d, *J* = 2.5 Hz, 1H), 7.44 (ddd, *J* = 8.9, 6.7, 1.1 Hz, 1H), 7.04 (td, *J* = 6.8, 1.4 Hz, 1H), 4.27–4.15 (m, 1H), 3.79–3.64 (m, 1H), 2.73 (d, *J* = 4.6 Hz, 3H, CONHCH_3_), 1.82–1.50 (m, 6H), 1.46–1.30 (m, 2H). ^13^C NMR (100 MHz, DMSO-*d*_6_) δ 167.36, 162.92, 142.88, 142.15, 140.43, 137.49, 137.44, 129.85, 129.68, 128.73, 126.85, 119.32, 114.05, 106.75, 106.26, 55.27, 48.86, 28.66, 28.18, 26.74, 22.84, 21.82.

2-(((1*R*,2*S*)-2-(2-(1H-imidazol-4-yl)acetamido)cyclohexyl)amino)-5-bromo-*N*-methyl-3-nitrobenzamide (**A4**)

Yellow powder, 64% yield, mp: 102–104 °C. ESI-MS: *m*/*z* 479.42 (M + H)^+^, 481.15 (M + 3H)^+^, C_19_H_23_BrN_6_O_4_ (478.10). ^1^NMR (400 MHz, DMSO-*d*_6_) δ 8.84 (d, *J* = 5.0 Hz, 1H), 8.15 (d, *J* = 2.4 Hz, 1H), 8.04 (t, *J* = 7.0 Hz, 2H), 7.68–7.57 (m, 2H), 6.84 (s, 1H), 3.98 (s, 1H), 3.55 (s, 2H), 3.24 (s, 2H, CH_2_CONH), 2.74 (d, *J* = 4.5 Hz, 3H, CONHCH_3_), 1.67–1.40 (m, 6H), 1.34–1.22 (m, 2H). ^13^C NMR (100 MHz, DMSO-*d*_6_) δ 169.91, 167.46, 142.36, 137.81, 136.87, 135.26, 132.80, 129.78, 128.91, 106.22, 55.39, 54.95, 34.52, 28.95, 28.22, 26.73.

*N*-((1*S*,2*R*)-2-((4-bromo-2-(methylcarbamoyl)-6-nitrophenyl)amino)cyclohexyl)nicotinamide (**A5**)

Yellow powder, 83% yield, mp: 178–180 °C. ESI-MS: *m*/*z* 476.85 (M + H)^+^, 478.12 (M + 3H)^+^, C_20_H_22_BrN_5_O_4_ (475.09). ^1^H NMR (400 MHz, DMSO-*d*_6_) δ 8.81 (d, *J* = 2.2 Hz, 1H), 8.67 (dd, *J* = 4.8, 1.6 Hz, 1H), 8.65–8.59 (m, 1H), 8.31 (d, *J* = 8.5 Hz, 1H), 8.15–8.07 (m, 2H), 7.99 (dt, *J* = 7.9, 2.0 Hz, 1H), 7.66 (d, *J* = 2.5 Hz, 1H), 7.46 (dd, *J* = 7.9, 4.8 Hz, 1H), 4.19 (s, 1H), 3.73–3.59 (m, 1H), 2.72 (d, *J* = 4.5 Hz, 3H, CONHCH_3_), 1.80–1.47 (m, 6H), 1.43–1.26 (m, 2H). ^13^C NMR (100 MHz, DMSO-*d*_6_) δ 167.31, 166.13, 152.02, 149.12, 142.61, 137.59, 137.42, 135.79, 130.86, 129.91, 128.63, 123.55, 106.43, 55.09, 49.48, 28.55, 28.03, 26.73, 22.96, 21.46.

*N*-((1*S*,2*R*)-2-((4-bromo-2-(methylcarbamoyl)-6-nitrophenyl)amino)cyclohexyl)-5-hydroxynicotinamide (**A6**)

Orange powder, 70% yield, mp: 138–140 °C. ESI-MS: *m*/*z* 492.73 (M + H)^+^, 494.12 (M + 3H)^+^, C_20_H_22_BrN_5_O_5_ (491.08). ^1^H NMR (400 MHz, DMSO-*d*_6_) δ 10.15 (s, 1H, OH), 8.63 (q, *J* = 4.5 Hz, 1H), 8.29 (d, *J* = 1.8 Hz, 1H), 8.27–8.20 (m, 2H), 8.13 (d, *J* = 2.4 Hz, 1H), 8.09 (d, *J* = 10.3 Hz, 1H), 7.66 (d, *J* = 2.5 Hz, 1H), 7.39 (t, *J* = 2.3 Hz, 1H), 4.18 (s, 1H), 3.76–3.56 (m, 1H), 2.73 (d, *J* = 4.5 Hz, 3H, CONHCH_3_), 1.80–1.47 (m, 6H), 1.44–1.26 (m, 2H). ^13^C NMR (100 MHz, DMSO-*d*_6_) δ 167.34, 165.92, 153.77, 142.50, 139.89, 139.40, 137.60, 137.39, 131.83, 129.90, 128.66, 122.18, 106.43, 60.24, 55.04, 28.59, 28.05, 26.73, 21.24.

*N*-((1*S*,2*R*)-2-((4-bromo-2-(methylcarbamoyl)-6-nitrophenyl)amino)cyclohexyl)-2-oxo-1,2-dihydropyridine-3-carboxamide (**A7**)

Yellow powder, 65% yield, mp: 156–159 °C. ESI-MS: *m*/*z* 492.39 (M + H)^+^, 494.13 (M + 3H)^+^, C_20_H_22_BrN_5_O_5_ (491.08). ^1^H NMR (400 MHz, DMSO-*d*_6_) δ 10.21 (d, *J* = 9.3 Hz, 1H), 8.65 (q, *J* = 4.5 Hz, 1H), 8.21 (dd, *J* = 7.1, 2.2 Hz, 1H), 8.12 (d, *J* = 2.4 Hz, 1H), 7.93 (d, *J* = 10.1 Hz, 1H), 7.71 (dd, *J* = 6.4, 2.3 Hz, 1H), 7.64 (d, *J* = 2.5 Hz, 1H), 6.52–6.43 (m, 1H), 4.26–4.15 (m, 1H), 3.65–3.51 (m, 1H), 2.73 (d, *J* = 4.5 Hz, 3H, CONHCH_3_), 1.82–1.46 (m, 5H), 1.44–1.27 (m, 3H). ^13^C NMR (100 MHz, DMSO-*d*_6_) δ 167.41, 163.42, 162.90, 144.33, 142.28, 139.83, 137.58, 137.40, 129.76, 120.47, 106.83, 106.60, 55.54, 48.00, 29.63, 28.32, 26.75, 23.85, 21.04.

*N*-((1*S*,2*R*)-2-((4-bromo-2-(methylcarbamoyl)-6-nitrophenyl)amino)cyclohexyl)-1H-indazole-5-carboxamide (**A8**)

Yellow powder, 72% yield, mp: 166–168 °C. ESI-MS: *m*/*z* 515.34 (M + H)^+^, 517.16 (M + 3H)^+^, C_22_H_23_BrN_6_O_4_ (514.10). ^1^H NMR (400 MHz, DMSO-*d*_6_) δ 13.18 (s, 1H), 8.62 (q, *J* = 4.6 Hz, 1H), 8.19 (d, *J* = 1.9 Hz, 2H), 8.17 (s, 1H), 8.12 (d, *J* = 2.4 Hz, 1H), 7.69–7.62 (m, 2H), 7.41–7.30 (m, 2H), 4.31–4.21 (m, 1H), 3.79–3.67 (m, 1H), 2.73 (d, *J* = 4.5 Hz, 3H), 1.84–1.47 (m, 6H), 1.47–1.29 (m, 2H). ^13^C NMR (100 MHz, DMSO-*d*_6_) δ 167.38, 167.28, 142.71, 140.57, 137.63, 137.27, 134.23, 129.94, 128.18, 125.53, 121.26, 120.49, 113.30, 106.28, 55.02, 49.51, 28.47, 28.23, 26.77.

*N*-((1*S*,2*R*)-2-((4-bromo-2-(methylcarbamoyl)-6-nitrophenyl)amino)cyclohexyl)-2-oxo-1,2-dihydroquinoline-4-carboxamide (**A9**)

Yellow powder, 68% yield, mp: 268–270 °C. ESI-MS: *m*/*z* 542.13 (M + H)^+^, C_24_H_24_BrN_5_O_5_ (541.10). ^1^H NMR (400 MHz, DMSO-*d*_6_) δ 11.90 (s, 1H, dihydroquinoline-CONH), 8.72 (t, *J* = 5.7 Hz, 2H), 8.28 (d, *J* = 10.0 Hz, 1H), 8.17 (d, *J* = 2.4 Hz, 1H), 7.72 (d, *J* = 2.6 Hz, 1H), 7.51 (t, *J* = 7.8 Hz, 2H), 7.32 (d, *J* = 8.3 Hz, 1H), 7.14 (t, *J* = 7.6 Hz, 1H), 6.46 (s, 1H), 4.34–4.21 (m, 1H), 3.77–3.62 (m, 1H), 2.77 (d, *J* = 4.5 Hz, 3H, CONHCH_3_), 1.77–1.46 (m, 6H), 1.45–1.26 (m, 2H). ^13^C NMR (100 MHz, DMSO-*d*_6_) δ 167.42, 166.41, 161.81, 146.49, 142.23, 139.52, 137.75, 137.11, 131.11, 130.02, 128.14, 126.30, 122.39, 120.38, 116.70, 115.95, 106.29, 54.65, 48.94, 28.04, 26.80.

*Tert*-butyl ((1*R*,2*S*)-2-((2-nitrophenyl)amino)cyclohexyl)carbamate (**6**)

1-Fluoro-2-nitrobenzene (0.8 g, 5.60 mmol), tert-butyl ((1*R*,2*S*)-2-aminocyclohexyl)carbamate (1.0 g, 4.67 mmol), and DIPEA (1.2 g, 9.34 mmol) were added to DMF and stirred at 50 °C for 3 h. The reaction was quenched by the addition of 30 mL of water, followed by extraction with CH_2_Cl_2_ (3 × 20 mL). The organic phases were combined, washed with saturated sodium chloride (3 × 30 mL), and dried over anhydrous sodium sulfate to yield the crude product, which was then purified by flash column chromatography on silica gel to yield intermediate **6**. Yellow powder, 82% yield, mp: 124–126 °C. ESI-MS: *m*/*z* 336.12 (M + H)^+^, C_17_H_25_N_3_O_4_ (335.18). ^1^H NMR (400 MHz, DMSO-*d*_6_) δ 8.23 (d, *J* = 8.9 Hz, 1H), 8.05 (dd, *J* = 8.8, 1.6 Hz, 1H), 7.49 (t, *J* = 7.7 Hz, 1H), 7.08 (dd, *J* = 20.9, 8.7 Hz, 2H), 6.66 (t, *J* = 7.7 Hz, 1H), 4.01 (s, 1H), 3.85 (s, 1H), 1.78–1.50 (m, 6H), 1.45–1.28 (m, 10H), 1.17 (s, 1H).

*Tert*-butyl ((1*R*,2*S*)-2-((2-aminophenyl)amino)cyclohexyl)carbamate (**7**)

A solution of ammonium chloride (NH_4_Cl, 1.59 g, 29.8 mmol) and intermediate 7 (1.0 g, 2.98 mmol) in tetrahydrofuran (THF) was added to a mixture of zinc powder (Zn, 1.95 g, 29.8 mmol) dissolved in a THF/H_2_O (1:2). The reaction was stirred at room temperature for 8 h. After the completion of the reaction, the reaction mixture was filtered through a layer of diatomaceous earth, and the filtrate was extracted with dichloromethane (3 × 10 mL), followed by washing with saturated sodium chloride solution and drying over anhydrous sodium sulfate. Purification by flash column chromatography on silica gel afforded intermediate **7**. White powder, 78% yield, mp: 146–148 °C. ESI-MS: *m*/*z* 306.39 (M + H)^+^, C_17_H_27_N_3_O_2_ (305.21). ^1^H NMR (400 MHz, DMSO-*d*_6_) δ 6.59 (dd, *J* = 7.4, 1.6 Hz, 1H), 6.57–6.49 (m, 1H), 6.47 (dd, *J* = 5.7, 1.7 Hz, 1H), 6.42 (ddd, *J* = 9.0, 5.8, 2.2 Hz, 1H), 4.46 (s, 2H), 3.89 (d, *J* = 7.9 Hz, 1H), 3.70 (s, 1H), 3.55 (s, 1H), 1.83–1.15 (m, 17H).

*N*^1^-((1*S*,2*R*)-2-aminocyclohexyl)benzene-1,2-diamine (**8**)

A solution of intermediate **7** in CH_2_Cl_2_ was treated with the addition of 3 mL of trifluoroacetic acid. The mixture was stirred at room temperature for 2 h, after which the pH was adjusted to 8–9 with a saturated sodium bicarbonate solution. Subsequent extraction with CH_2_Cl_2_ (3 × 10 mL) was performed, followed by washing the organic layer with saturated sodium chloride (3 × 20 mL) and drying over anhydrous sodium sulfate to yield the crude product **8**. This crude material was used directly without further purification.

2-(((1*R*,2*S*)-2-((2-Aminophenyl)amino)cyclohexyl)amino)-5-bromo-*N*-methyl-3-nitrobenzamide (**9**)

Intermediate **8** (0.50 g, 2.44 mmol), intermediate **2** (0.81 g, 2.93 mmol), and DIPEA (0.63 g, 4.88 mmol) were added to DMF and stirred at 50 °C for 3 h. The reaction was quenched by the addition of 30 mL of water, followed by extraction with CH_2_Cl_2_ (3 × 20 mL). The organic phases were combined, washed with saturated sodium chloride (3 × 30 mL), and dried over anhydrous sodium sulfate to yield the crude product, which was then purified by flash column chromatography on silica gel to yield intermediate **9**. Yellow powder, 70% yield, mp: 78–80 °C. ESI-MS: *m*/*z* 462.41 (M + H)^+^, 464.35 (M + 3H)^+^, C_20_H_24_BrN_5_O_3_ (461.11).

General synthesis procedure for compounds **A10**–**A15**

A solution of various carboxylic acids (1.2 eq), HATU (1.5 eq), and DIPEA (2.0 eq) was prepared in DMF. The mixture was stirred at 0 °C for 30 min, after which intermediate **9** (1.0 eq) was added. The reaction mixture was then stirred at room temperature for 3 to 8 h. The reaction was terminated by the addition of 30 mL of water, followed by extraction with CH_2_Cl_2_ (3 × 20 mL). The organic phases were combined, washed with saturated sodium chloride (3 × 30 mL), and dried over anhydrous sodium sulfate to yield the crude product. Subsequent purification by flash column chromatography on silica gel afforded the target compounds **A10**–**A15**.

5-Bromo-2-(((1*R*,2*S*)-2-((2-(2-hydroxybenzamido)phenyl)amino)cyclohexyl)amino)-*N*-methyl-3-nitrobenzamide (**A10**)

Yellow powder, 69% yield, mp: 118–120 °C. ESI-MS: *m*/*z* 582.20 (M + H)^+^, 584.12 (M + 3H)^+^, C_27_H_28_BrN_5_O_5_ (581.13). ^1^H NMR (400 MHz, DMSO-*d*_6_) δ 10.45 (s, 1H, OH), 8.68 (s, 1H, PhCONHPh), 8.25 (d, *J* = 9.8 Hz, 1H), 8.00 (d, *J* = 2.5 Hz, 1H), 7.90 (d, *J* = 7.8 Hz, 1H), 7.55 (d, *J* = 2.5 Hz, 1H), 7.38 (t, *J* = 7.7 Hz, 1H), 7.30 (d, *J* = 7.8 Hz, 1H), 7.03 (t, *J* = 7.7 Hz, 1H), 6.91 (d, *J* = 8.3 Hz, 1H), 6.85 (t, *J* = 7.5 Hz, 1H), 6.76 (d, *J* = 8.2 Hz, 1H), 6.68 (t, *J* = 7.5 Hz, 1H), 4.62 (d, *J* = 9.3 Hz, 1H), 3.80–3.70 (m, 1H), 3.69–3.61 (m, 1H), 2.65 (d, *J* = 4.5 Hz, 3H, CONHCH_3_), 1.76–1.43 (m, 6H), 1.42–1.26 (m, 2H). ^13^C NMR (100 MHz, DMSO-*d*_6_) δ 167.52, 167.31, 142.14, 137.72, 136.51, 133.85, 129.88, 129.65, 127.19, 126.76, 124.92, 117.94, 117.55, 117.45, 113.52, 106.03, 55.07, 52.38, 28.54, 28.37, 26.64.

*N*-(2-(((1*S*,2*R*)-2-((4-bromo-2-(methylcarbamoyl)-6-nitrophenyl)amino)cyclohexyl)amino)phenyl)nicotinamide (**A11**)

Yellow powder, 87% yield, mp: 108–110 °C. ESI-MS: *m*/*z* 567.90 (M + H)^+^, 569.04 (M + 3H)^+^, C_26_H_27_BrN_6_O_4_(566.13). ^1^H NMR (400 MHz, DMSO-*d*_6_) δ 10.11 (s, 1H, PhCONHPh), 9.07 (d, *J* = 2.2 Hz, 1H), 8.83–8.70 (m, 1H), 8.55 (d, *J* = 5.1 Hz, 1H), 8.34 (d, *J* = 9.5 Hz, 1H), 8.24 (d, *J* = 7.9 Hz, 1H), 8.02 (d, *J* = 2.4 Hz, 1H), 7.62–7.49 (m, 2H), 7.20 (d, *J* = 7.7 Hz, 1H), 7.09 (t, *J* = 7.7 Hz, 1H), 6.81 (d, *J* = 8.2 Hz, 1H), 6.71 (t, *J* = 7.5 Hz, 1H), 4.76 (d, *J* = 9.6 Hz, 1H), 3.76 (s, 1H), 3.65 (s, 1H), 2.67 (d, *J* = 4.5 Hz, 3H, CONHCH_3_), 1.82–1.43 (m, 6H), 1.43–1.22 (m, 2H). ^13^C NMR (100 MHz, DMSO-*d*_6_) δ 167.47, 164.79, 152.53, 149.23, 142.51, 141.97, 137.85, 136.39, 135.88, 130.39, 129.85, 127.59, 127.07, 125.14, 123.87, 117.51, 113.89, 106.00, 55.04, 52.30, 28.70, 28.10, 26.67.

2-(((1*R*,2*S*)-2-((2-(2-(1H-imidazol-4-yl)acetamido)phenyl)amino)cyclohexyl)amino)-5-bromo-*N*-methyl-3-nitrobenzamide (**A12**)

Yellow powder, 72% yield, mp: 128–130 °C. ESI-MS: *m*/*z* 570.76 (M + H)^+^, 572.16 (M + 3H)^+^, C_25_H_28_BrN_7_O_4_ (569.14). ^1^H NMR (400 MHz, DMSO-*d*_6_) δ 11.93 (s, 1H, imidazole-NH), 9.46 (s, 1H), 8.66 (d, *J* = 5.0 Hz, 1H), 8.26 (d, *J* = 9.6 Hz, 1H), 8.11 (d, *J* = 2.5 Hz, 1H), 7.63 (d, *J* = 2.5 Hz, 1H), 7.55–7.45 (m, 1H), 7.10 (dd, *J* = 7.8, 1.6 Hz, 1H), 6.96 (d, *J* = 7.9 Hz, 1H), 6.93 (s, 1H), 6.66 (d, *J* = 8.2 Hz, 1H), 6.60 (t, *J* = 7.5 Hz, 1H), 4.67 (d, *J* = 9.4 Hz, 1H), 3.73 (s, 1H), 3.63 (s, 1H), 3.62–3.47 (m, 2H, CONH_2_-imidazole), 2.71 (d, *J* = 4.5 Hz, 3H, CONHCH_3_), 1.75–1.60 (m, 2H), 1.56–1.39 (m, 4H), 1.24 (s, 2H). ^13^C NMR (100 MHz, DMSO-*d*_6_) δ 169.57, 167.51, 142.02, 137.95, 136.45, 135.33, 129.84, 128.88, 126.84, 126.23, 125.17, 117.08, 113.10, 106.03, 55.12, 51.94, 35.68, 28.47, 28.17, 26.73.

*N*-(2-(((1*S*,2*R*)-2-((4-bromo-2-(methylcarbamoyl)-6-nitrophenyl)amino)cyclohexyl)amino)phenyl)pyrazine-2-carboxamide (**A13**)

Yellow powder, 85% yield, mp: 156–158 °C. ESI-MS: *m*/*z* 568.49 (M + H)^+^, 570.16 (M + 3H)^+^, C_25_H_26_BrN_7_O_4_ (567.12). ^1^H NMR (400 MHz, DMSO-*d*_6_) δ 10.21 (s, 1H), 9.22 (s, 1H), 8.92 (d, *J* = 2.5 Hz, 1H), 8.72 (d, *J* = 2.3 Hz, 1H), 8.47 (s, 1H), 8.37 (d, *J* = 9.5 Hz, 1H), 7.96 (d, *J* = 2.5 Hz, 1H), 7.55 (d, *J* = 2.5 Hz, 1H), 7.39 (d, *J* = 7.8 Hz, 1H), 7.07 (t, *J* = 7.8 Hz, 1H), 6.83 (d, *J* = 8.2 Hz, 1H), 6.74 (t, *J* = 7.6 Hz, 1H), 4.73 (d, *J* = 8.9 Hz, 1H), 3.70 (s, 1H), 3.64 (s, 1H), 2.64 (d, *J* = 4.4 Hz, 3H, CONHCH_3_), 1.79–1.22 (m, 8H). ^13^C NMR (100 MHz, DMSO-*d*_6_) δ 167.37, 162.20, 148.07, 145.28, 144.26, 143.69, 142.34, 141.69, 137.80, 136.35, 129.85, 128.12, 127.15, 126.15, 125.52, 118.16, 114.90, 105.86, 54.85, 52.79, 28.53, 28.06, 26.65, 22.62, 21.62.

5-Bromo-2-(((1*R*,2*S*)-2-((2-(2-fluorobenzamido)phenyl)amino)cyclohexyl)amino)-*N*-methyl-3-nitrobenzamide (**A14**)

Yellow powder, 86% yield, mp: 178–180 °C. ESI-MS: *m*/*z* 584.22 (M + H)^+^, 586.14 (M + 3H)^+^, C_27_H_27_BrFN_5_O_4_ (583.12). ^1^H NMR (400 MHz, DMSO-*d*_6_) δ 9.95 (s, 1H), 8.62 (d, *J* = 5.3 Hz, 1H), 8.39 (d, *J* = 9.4 Hz, 1H), 8.08 (d, *J* = 2.5 Hz, 1H), 7.68 (t, *J* = 7.4 Hz, 1H), 7.61 (d, *J* = 2.6 Hz, 1H), 7.59–7.53 (m, 1H), 7.36–7.28 (m, 2H), 7.24 (d, *J* = 7.7 Hz, 1H), 7.05 (t, *J* = 7.9 Hz, 1H), 6.77 (d, *J* = 8.2 Hz, 1H), 6.69 (t, *J* = 7.4 Hz, 1H), 4.75 (d, *J* = 9.7 Hz, 1H), 3.79 (s, 1H), 3.66 (s, 1H), 2.70 (d, *J* = 4.5 Hz, 3H, CONHCH_3_), 1.82–1.23 (m, 8H). ^13^C NMR (100 MHz, DMSO-*d*_6_) δ 167.52, 163.51, 159.57 (d, ^1^*J_CF_* = 249.4 Hz, 37C), 142.14, 141.88, 137.97, 136.25, 133.05, 132.97, 130.49, 129.80, 128.72, 127.39, 126.67, 125.07, 124.93, 124.86, 124.71, 117.49, 116.72, 116.50, 113.73, 105.96, 55.13, 51.94, 28.37, 26.72, 23.38.

*N*-(2-(((1*S*,2*R*)-2-((4-bromo-2-(methylcarbamoyl)-6-nitrophenyl)amino)cyclohexyl)amino)phenyl)-1H-pyrazole-4-carboxamide (**A15**)

Yellow powder, 83% yield, mp: 150–152 °C. ESI-MS: *m*/*z* 556.82 (M + H)^+^, 558.18 (M + 3H)^+^, C_24_H_26_BrN_7_O_4_ (555.12). ^1^H NMR (400 MHz, DMSO-*d*_6_) δ 13.21 (s, 1H, pyrazole-NH), 9.59 (s, 1H, PhNHCO), 8.56 (d, *J* = 4.8 Hz, 1H), 8.34 (d, *J* = 9.4 Hz, 1H), 8.28 (s, 1H), 8.06 (d, *J* = 2.5 Hz, 1H), 7.98 (s, 1H), 7.57 (d, *J* = 2.5 Hz, 1H), 7.12 (dd, *J* = 7.7, 1.5 Hz, 1H), 7.04 (t, *J* = 7.7 Hz, 1H), 6.77 (d, *J* = 8.3 Hz, 1H), 6.68 (t, *J* = 7.5 Hz, 1H), 4.83 (d, *J* = 9.7 Hz, 1H), 3.74 (s, 1H), 3.64 (s, 1H), 2.67 (d, *J* = 4.5 Hz, 3H, CONHCH_3_), 1.79–1.42 (m, 6H), 1.24 (s, 2H). ^13^C NMR (100 MHz, DMSO-*d*_6_) δ 167.51, 161.85, 142.82, 141.92, 138.00, 136.10, 129.78, 127.18, 126.99, 125.65, 117.76, 117.56, 113.99, 105.89, 55.13, 52.35, 28.64, 28.33, 26.67.

### 4.3. Enzymatic Assay of SARS-CoV-2 Main Protease

The SARS-CoV-2 M^pro^/3CL^pro^ inhibitor screening kit (Beyotime, Beijing, China, P0312S) was used to evaluate the inhibitory activity of the target compounds against SARS-CoV-2 M^pro^ via a fluorescence resonance energy transfer (FRET) assay. The procedure was conducted in strict accordance with the kit’s instructions. The methodology is as follows: the enzyme stock solution was initially diluted 93-fold with assay buffer, and the 10 mM compound stock solution in DMSO was adjusted to the necessary concentrations with assay buffer. Then, in a 96-well black plate, 93 μL of enzyme, 5 μL of the compound, and 2 μL of the substrate (Dabcyl-KTSAVLQSGFRKME-Edans) were sequentially added. After incubation at 37 °C in the dark for 5 min, the fluorescence intensities (RFU, Relative Fluorescence Units) were promptly measured for each well using a SpectraMax iD5 plate reader (Molecular Devices, San Jose, CA, USA) with excitation at 340 nm and emission at 490 nm. A blank control without enzyme was also established. Compounds were screened initially at a concentration of 1 μM, and those with inhibition rates exceeding 50% were assessed for their EC_50_ values. IC_50_ values were calculated using GraphPad Prism 8 software. Triplicate measurements were performed for each sample.

### 4.4. Anti-SARS-CoV-2 Assay

#### 4.4.1. Cells and Virus

VeroE6-eGFP cells (provided by M. van Loock, Janssen Pharmaceutica, Beerse, Belgium) were maintained in DMEM (Gibco, cat no 41965-039, Waltham, MA, USA) supplemented with heat-inactivated 10% FBS and 500 µg/mL Geneticin (Gibco, cat no 10131-0275, Waltham, MA, USA) and kept in a humidified 5% CO_2_ incubator at 37 °C. For the production of virus stocks and during virus experiments, the cells were maintained in assay medium. The VeroE6-eGFP assay medium consisted of DMEM supplemented with 2% FBS. The SARS-CoV-2 isolate used in this study was the BetaCov/Belgium/GHB-03021/2020 (EPI ISL407976|2020-02-03), which was isolated from a Belgian patient returning from Wuhan in February 2020. Virus stocks were inoculated and passaged first in HuH-7 cells and then passaged 7 times on VeroE6-eGFP cells. GHB-03021 has a ∆TQTNS deletion at 676–680 residues that is typical for SARS-CoV-2 strains that have been passaged several times on VeroE6 cells [29].

#### 4.4.2. Antiviral Assay

VeroE6-GFP cells were seeded at a density of 25,000 cells/well in 96-well plates (Greiner Bio One, catalogue no. 655090, Kremsmünster, Austria) and pre-treated with three-fold serial dilutions of the compounds overnight (37 °C, 5% CO_2_, and 95% relative humidity) in the presence of CP-100356 (a P-glycoprotein inhibitor) at a final concentration 0.5 µM. On the next day (day 0), cells were infected with the SARS-CoV-2 inoculum at a multiplicity of infection (MOI) of 0.001 median tissue culture infectious dose (TCID50) per cell. After a four-day incubation, the expression of eGFP was evaluated using an argon laser-scanning microscope with excitation at 488 nm and emission at 510 nm. The fluorescence images, as determined by high-content imaging (HCI), were translated into signal values. Percentage of inhibition was calculated by subtracting the background (number of fluorescent pixels in the untreated–infected control wells) and normalizing to the untreated–uninfected control wells (also background subtracted). The 50% effective concentration (EC_50_, the concentration of compound required to reduce virus-induced eGFP signals by 50% relative to uninfected control cells) was determined using logarithmic interpolation. Cytotoxicity of compounds was assessed in a similar set-up in treated–uninfected cultures, where metabolic activity was quantified at day 5 using the MTS assay as described earlier [30]. The 50% cytotoxic concentration (CC_50_, the concentration at which cell viability reduced to 50%) was also calculated by logarithmic interpolation.

### 4.5. Molecular Modeling Studies

The SARS-CoV-2 M^pro^ (PDB code: 7EN8) protein complex structure was downloaded from the PDB databank (rcsb.org, accessed on 12 January 2025). The structures of protein and molecular ligand (**A5**, **A6** and **A9**) were prepared as described previously [32]. A maximum 5 conformations were generated per ligand, and the highest-scoring conformations (Ligprep energy) were selected for docking. All other parameters were set as default. The protein binding pocket was identified by the original cocrystallized ligands using the Receptor Grid Generation module. The binding conformations of each molecular with the best docking score were selected for analysis and MD simulations.

A 200 ns molecular dynamics (MD) simulation was conducted for the binding poses of compounds **A5**, **A6**, and **A9** with SARS-CoV-2 M^pro^ using the Desmond module from the Schrödinger suite 2023-1. The simulation environment for the protein–ligand complex was explicitly solvated with the TIP3P water model within an orthorhombic box, maintaining a 10 Å buffer zone. The system was neutralized with Na^+^ or Cl^−^ and adjusted with an additional 0.15 M NaCl. Prior to the simulation, the system was energy-minimized and then subjected to a 200 ns MD simulation under NPT conditions at 300 K and 1.013 bar, as facilitated by the Desmond module of the Schrödinger suite 2023-1. Post-simulation analysis was performed using the simulation interactions diagram module to generate the protein backbone and ligand heavy atom RMSDs, as well as RMSF plots. The binding energy (MM/GBSA) between **A5**, **A6**, and **A9** with SARS-CoV-2 M^pro^ were calculated over the 200 ns period using the MM/GBSA module based on the MD simulations. It took a Desmond trajectory file, split it into individual snapshots, ran the Prime-MMGBSA calculations on each frame (2 ns), and yielded the average calculated binding energy.

## 5. Conclusions

In this study, we employed a fragment-based multilevel virtual screening strategy to further study the structure–activity relationship (SAR) of the S1 pocket-targeting group in **WU-04** and enhance its antiviral activity. Through a multilevel virtual screening and feasibility analysis of a newly constructed small molecule compound library, we identified 14 structurally novel compounds and one previously reported small molecule. Among them, compounds **A5**, **A6**, and **A9** were identified as promising lead compounds with strong enzymatic inhibitory activities against SARS-CoV-2 M^pro^. Notably, **A9** demonstrated robust antiviral activity at the cellular level, with an EC_50_ value of 0.18 μM, comparable to the marketed drug Nirmatrelvir (EC_50_ = 0.123 μM) and slightly weaker than **WU-04** (EC_50_ = 0.042 μM). Molecular dynamics simulations provided mechanistic insights into the interactions between these compounds and the target protein’s binding pocket, further validating their potential as SARS-CoV-2 inhibitors. Our findings highlight the effectiveness of the fragment-based multilevel virtual screening strategy in the discovery of novel anti- SARS-CoV-2 inhibitors. Compound **A9**, in particular, represents a promising lead for further optimization.

## Figures and Tables

**Table 1:** Inhibitory activities of **A5**, **A6** and **A9** against SARS-CoV-2 M^pro^.

Compounds	IC_50_ (μM) ^1^
**A5**	1.05 ± 0.067
**A6**	1.08 ± 0.023
**A9**	0.154 ± 0.006
**WU-04**	0.125 ± 0.002
Nirmatrelvir	0.103 ± 0.001
Ensitrelvir	0.113 ± 0.002

^1^ The data represent the mean ± SD of three independent measurements.

**Table 2:** Antiviral activity and cytotoxicity of **A5**, **A6**, and **A9** against SARS-CoV-2.

Compounds	EC_50_ (μM) ^1^	CC_50_ (μM)	SI ^3^
**A5**	2.01 ± 0.34	>100	>49.7
**A6**	6.05 ± 0.16	>100	>16.5
**A9**	0.18 ± 0.02	>100	>555
**WU-04**	0.042 ± 0.022	>100	>2381
Nirmatrelvir	0.123 ± 0.034 ^2^	>100 ^2^	>813

^1^ The data represent the mean ± SD of two independent replicates. ^2^ The antiviral data of Nirmatrelvir were obtained from a previous report, which was measured under identical conditions [31]. ^3^ SI (Selection Index) = CC_50_/EC_50_.

**Table 3:** Overview of the observed inhibitory potencies of **A5**, **A6**, and **A9**, along with the representative contributions to the binding free energy estimation (MM/GBSA).

Compounds	IC_50_ (μM)	MM/GBSA (kcal/mol)				
		Coulomb	Hbond	Lipo	vdW	Total
**A5**	1.05	−18.76	−1.54	−13.08	−57.70	−67.45
**A6**	1.08	−24.44	−1.65	−9.7	−56.7	−64.15
**A9**	0.154	−26.04	−2.43	−13.98	−61.38	−73.97

## Data Availability

The data presented in this study are available on request from the corresponding author.

## Supplementary Materials

The following supporting information can be downloaded at: https://www.mdpi.com/article/10.3390/ijms26020670/s1.
