# Supplementary material for: Optimization of SARS-CoV-2 Mpro Inhibitors by a Structure-Based Multilevel Virtual Screening Method
Source: Int J Mol Sci. 2025 Jan 14;26(2):670. doi: 10.3390/ijms26020670 (PMC11765572; doi:10.3390/ijms26020670)
Supplement: Supplementary file 1 [file ijms-26-00670-s001.zip › ijms-3381021-supplementary.pdf]

Support Information

Optimization of SARS-CoV-2 M^{pro} Inhibitors by a Structure-Based Multilevel Virtual Screening Method

Lanlan Jing [1], Fabao Zhao [1], Lin Zheng [1], Bairu Meng [1], Shenghua Gao [1], Manon Laporte[2], Dirk Jochmans[2], Steven De Jonghe[3], Johan Neyts[2], Peng Zhan [1], Dongwei Kang [1,4,*] and Xinyong Liu [1,4,*]

[1]Key Laboratory of Chemical Biology (Ministry of Education), Department of Medicinal Chemistry, School of Pharmaceutical Sciences, Cheeloo College of Medicine, Shandong University, 44 West Culture Road, Jinan 250012, China

[2]Antiviral Drug & Vaccine Research Group, Department of Microbiology, Immunology and Transplantation, Rega Institute for Medical Research, KU Leuven, Herestraat 49, B-3000 Leuven, Belgium

[3]Molecular, Structural and Translational Virology Research Group, Department of Microbiology, Immunology and Transplantation, Rega Institute for Medical Research, KU Leuven, Herestraat 49, B-3000 Leuven, Belgium

[4]China-Belgium Collaborative Research Center for Innovative Antiviral Drugs of Shandong Province, Shandong University, 44 West Culture Road, Jinan 250012, China

[*]Correspondence: kangdongwei@sdu.edu.cn (D.K.); xinyongl@sdu.edu.cn (X.L.)

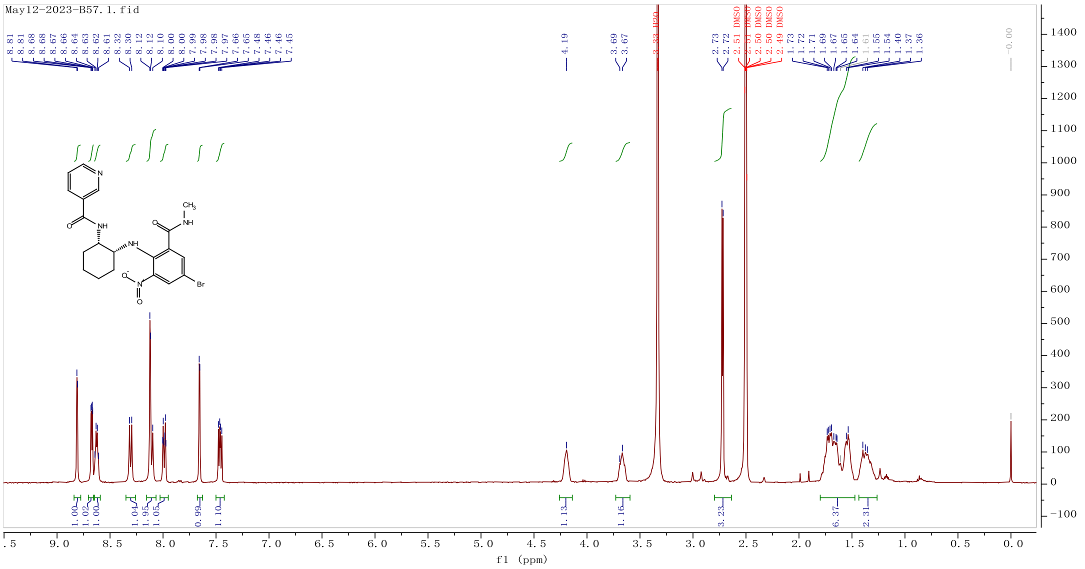

Figure S1: The ^{1}H NMR spectrum of **A5**.

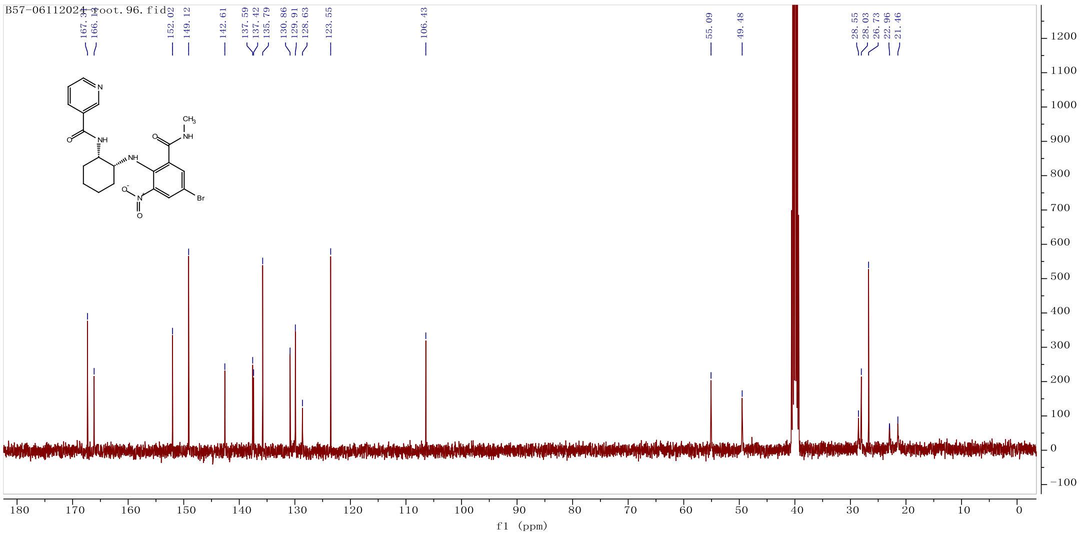

Figure S2: The ^{13}C NMR spectrum of **A5**.

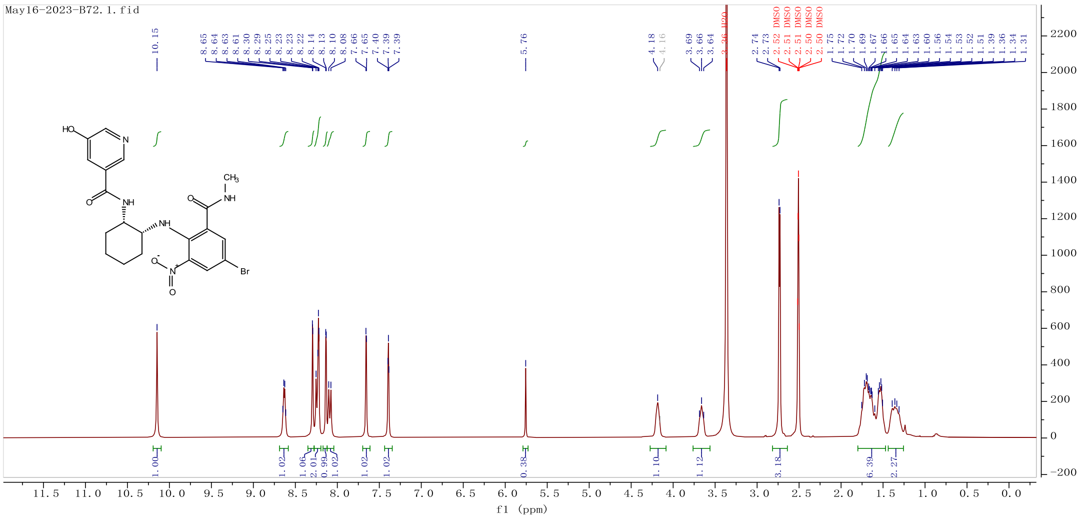

Figure S3: The ^{1}H NMR spectrum of **A6**.

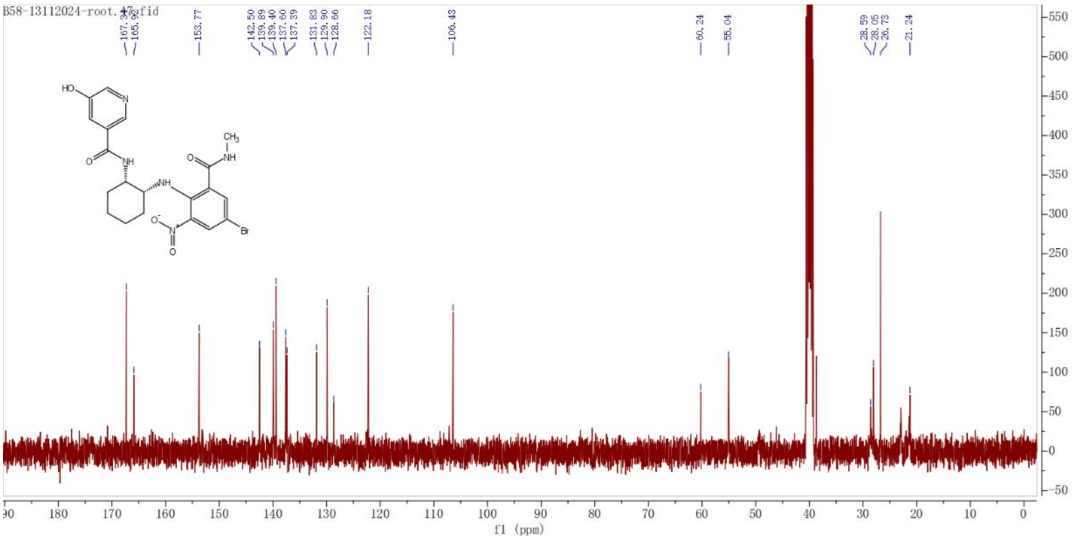

Figure S4: The ^{13}C NMR spectrum of **A6**.

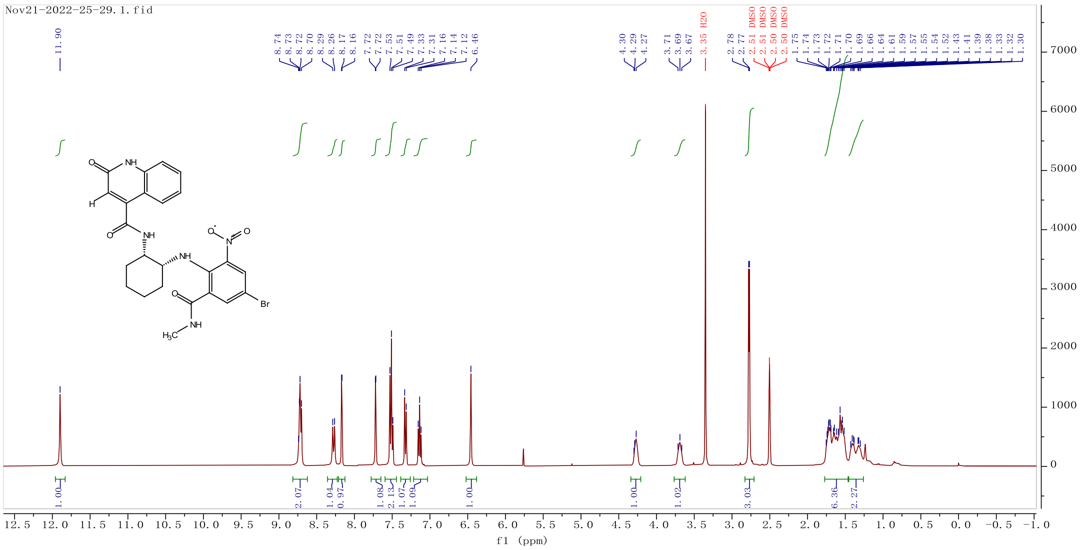

Figure S5: The ^{1}H NMR spectrum of **A9**.

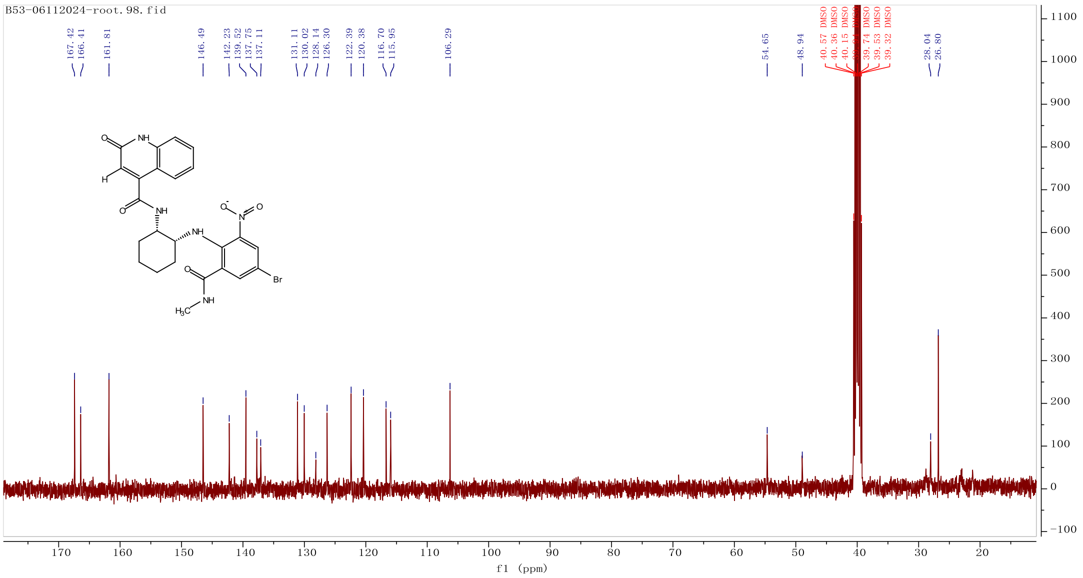

Figure S6: The ^{13}C NMR spectrum of **A9**.
